# The E3 ubiquitin ligase STUB1 inhibits Senecavirus A replication by mediating VP1 ubiquitination and proteasomal degradation

**DOI:** 10.1128/jvi.01152-25

**Published:** 2025-10-01

**Authors:** Penghui Zeng, Jingyu Mao, Jinshuo Guo, Xiaoyu Yang, Yongyan Shi, Xiaoyu Wang, Jiangwei Song, Jianwei Zhou, Lei Hou, Jue Liu

**Affiliations:** 1 College of Veterinary Medicine, Yangzhou University 38043, Yangzhou, China; 2 Jiangsu Key Laboratory of Zoonosis, Yangzhou University 38043, Yangzhou, China; 3 Beijing Key Laboratory for Prevention and Control of Infectious Diseases in Livestock and Poultry, Institute of Animal Husbandry and Veterinary Medicine, Beijing Academy of Agriculture and Forestry Sciences 107624, Beijing, China; University of Michigan Medical School, Ann Arbor, Michigan, USA

**Keywords:** SVA VP1 protein, STUB1, ubiquitination degradation, HSP70, HSC70, SVA 3Cpro

## Abstract

Senecavirus A (SVA), an emerging vesicular pathogen, poses a significant threat to the global pig industry. STIP1 homology and U-box-containing protein 1 (STUB1), a chaperone-dependent E3 ubiquitin ligase, plays a pivotal role in protein quality control by mediating target protein degradation. However, its precise role of STUB1 in regulating SVA replication remains undefined. In this study, we combined liquid chromatography-mass spectrometry, confocal imaging, and Western blotting to demonstrate that STUB1 interacts with the SVA VP1 protein and negatively regulates SVA replication. Mechanistically, STUB1 promotes the ubiquitination-dependent degradation of VP1 by specifically targeting lysine residues at positions 177 and 260 (K177 and K260). This degradation process is significantly enhanced by heat shock protein 70 (HSP70) and heat shock cognate protein 70 (HSC70), which strengthen the STUB1-VP1 interaction. Notably, the SVA 3C protease (3Cpro) counteracts this antiviral defense by enzymatically reducing STUB1 expression. *In vivo* studies using a mouse model showed that a VP1 mutant virus lacking STUB1-targeted ubiquitination sites replicates more efficiently than the wild-type strain, resulting in significantly higher viral loads across multiple tissues and more severe pulmonary pathology. Together, these findings reveal that STUB1 inhibits SVA replication through ubiquitination-dependent degradation of VP1, a process that is antagonized by viral 3C protease via suppression of STUB1 expression.

**IMPORTANCE:**

Viruses have evolved diverse strategies to enhance their replication efficiency. Senecavirus A (SVA), an emerging porcine pathogen associated with vesicular disease outbreaks, has become increasingly prevalent in swine populations worldwide. As a chaperone-dependent E3 ubiquitin ligase, STUB1 plays a crucial role in maintaining cellular protein homeostasis. In this study, we elucidated the functional interplay between STUB1 and SVA replication. Our results demonstrate that STUB1 directly interacts with the viral protein VP1 and mediates its ubiquitination-dependent degradation through specific targeting of lysine residues at positions 177 and 260 (K177 and K260), thereby significantly inhibiting viral replication. However, SVA has evolved a countermeasure, whereby its 3C protease (3Cpro) downregulates STUB1 expression, effectively blocking VP1 degradation and subverting this host antiviral defense to promote viral propagation. These findings not only reveal novel host-virus interaction mechanisms but also provide valuable molecular targets for developing innovative strategies to control SVA infection.

## INTRODUCTION

Senecavirus A (SVA), previously known as the Seneca Valley virus (SVV), is a single-stranded, positive-sense, non-enveloped RNA virus belonging to the genus Senecavirus within the *Picornaviridae* family (1). First identified as a cell culture contaminant in 2002, its complete genome shows a close relationship to that of *Cardiovirus* (1). The viral genome, approximately 7.2 kb in length, contains a single open reading frame (ORF) encoding a polyprotein. This polyprotein is proteolytically processed into structural proteins (VP1, VP2, VP3, and VP4) and nonstructural proteins (Lpro, 2A, 2B, 2C, 3A, 3B, 3C, and 3D) (1). SVA infections can cause vesicular diseases, leading to substantial economic losses in the global swine industry (2–4). Since the first detection of Chinese strains of SVA in 2015, increasing numbers of infections have been reported across various provinces in China, indicating widespread transmission (5, 6). Additionally, SVA has attracted attention as a potential oncolytic virus for cancer therapy (7, 8). However, no specific or effective treatment for SVA is currently available in clinical practice. A deeper understanding of the molecular mechanisms underlying viral replication is essential for developing effective strategies to prevent and control SVA infections.

The structural proteins of SVA are derived from the initially synthesized P1 polymeric protein. Once mature P1 is produced, it is cleaved by SVA 3C protease into VP1, VP3, and VP0; VP0 is subsequently cleaved into VP2 and VP4 (1, 9–11). Similar to other members of the *Picornaviridae* family, the antigenic regions of SVA are primarily composed of VP1 and VP3, with VP1 exhibiting the strongest antigenicity (12). Furthermore, the SVA VP1 protein collaborates with VP3 and 3Cpro to activate the AKT-AMPK-MAPK-mTOR signaling pathway (13), facilitating viral replication. It also plays a critical role in viral attachment and entry into host cells, making it a potential target for SVA intervention (14). The 3Cpro is a multifunctional protein involved in viral pathogenesis: it promotes viral protein maturation and suppresses host antiviral responses by cleaving histone deacetylase 4 (HDAC4) to inhibit type I interferon signaling and by degrading interferon regulatory factors 3 and 7 (IRF3 and IRF7) to disrupt the innate immune response (15, 16).

The ubiquitin–proteasome system is a highly efficient protein degradation pathway in eukaryotic cells, responsible for the selective degradation of proteins and crucial for maintaining cellular homeostasis (17). It contributes to antiviral defense by degrading viral proteins; for example, the host E3 ligase Hrd1 ubiquitinates and degrades the H protein of the canine distemper virus to inhibit viral replication (18). RNF5 restricts SARS-CoV-2 replication by targeting its envelope protein for degradation (19); and TRIM7 inhibits enterovirus replication by mediating the degradation of the enteroviral 2C protein (20).

STIP1 homology and U-box containing protein 1 (STUB1), formerly known as the C-terminus of HSC70-interacting protein (CHIP), is a chaperone-dependent E3 ubiquitin ligase (21). It is highly expressed in metabolically active tissues and regions prone to protein misfolding, such as the skeletal muscle, brain, and heart (22). The N-terminal domain of STUB1 interacts with heat shock proteins to enhance the resilience to stress (23), while its C-terminal U-box domain mediates the ubiquitination and subsequent degradation of specific substrate proteins (24). STUB1 has been implicated in various immune responses, including the regulation of immune cell differentiation, maturation, and inflammation (25–28). It is also involved in viral replication: for instance, STUB1 modulates antiviral RNA interference by inducing the ubiquitination and degradation of Dicer and AGO2 in mammals (29); mediates the degradation of the porcine deltacoronavirus nucleocapsid protein (30); and is targeted by the SUMO-interacting motif of EBNA1 to maintain the latency of Epstein–Barr virus (31). However, the role of STUB1-mediated ubiquitination in SVA infection remains unclear.

In this study, we demonstrate that STUB1 exerts antiviral activity by mediating ubiquitination-dependent degradation of VP1 through specific targeting of lysine residues at positions 177 and 260 (K177/K260). However, SVA infection counteracts this host defense mechanism through 3Cpro-mediated downregulation of STUB1 expression, thereby facilitating viral infection. Collectively, our results reveal a novel host-virus interaction mechanism that provides important insights for the prevention and control of SVA infection.

## RESULTS

### STUB1 interacts with SVA VP1

**Fig 1:**
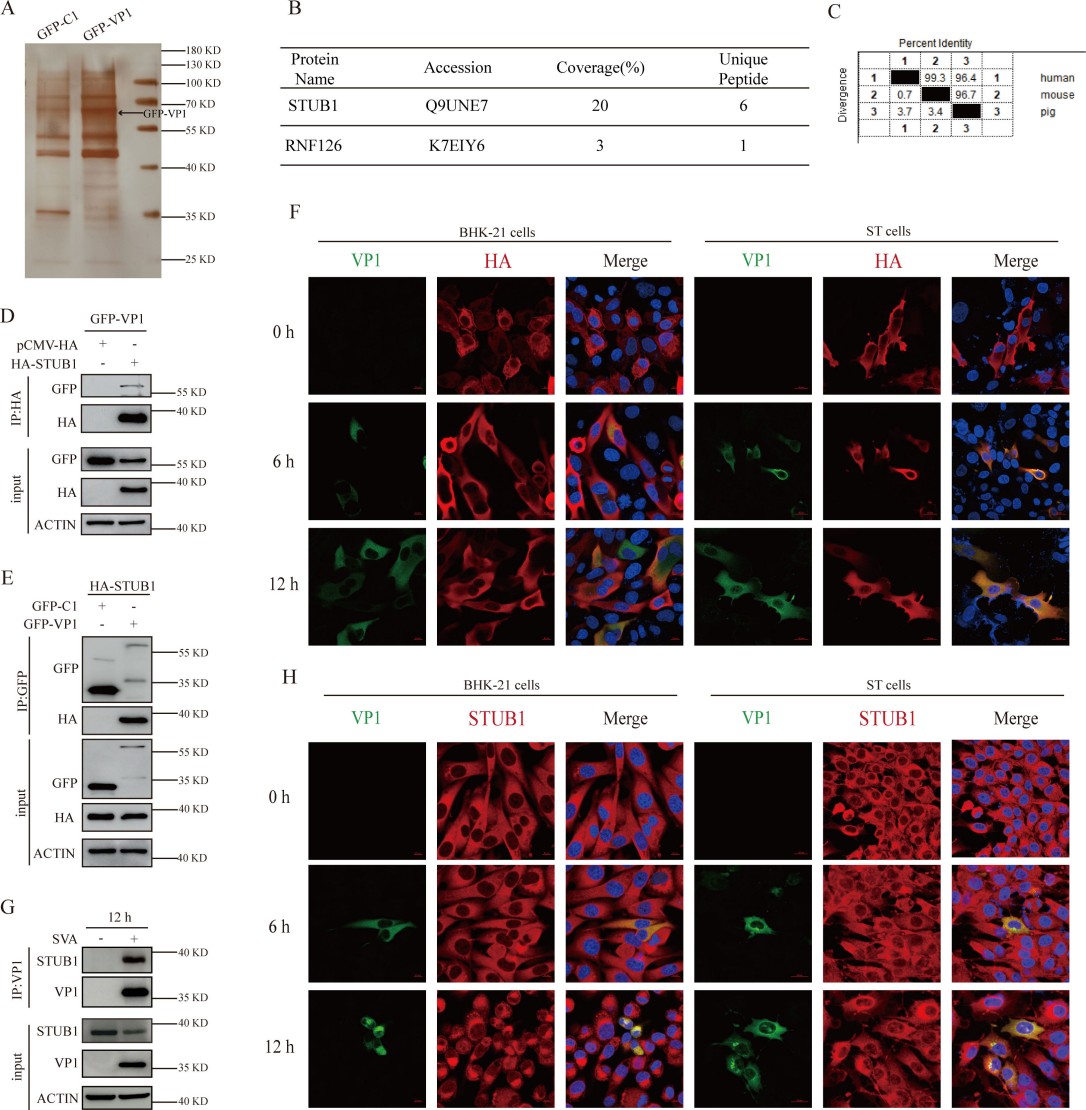
STUB1 interacts with SVA VP1 (**A**) Detection of GFP-VP1 expression in HEK-293T cells and visualization of VP1-interacting proteins via silver staining. GFP-C1 (control) or GFP-VP1 was transfected into HEK-293T cells; 24 h post-transfection, anti-GFP agarose beads were used to immunoprecipitate VP1 and its binding partners. (**B**) E3 ubiquitin ligases interacting with VP1 identified by liquid chromatography-tandem mass spectrometry. (**C**) Amino acid sequence alignment of STUB1 from porcine, mouse, and human using MegAlign. (**D–E**) Co-immunoprecipitation (co-IP) analysis of HEK-293T cells coexpressing HA-STUB1 and GFP-VP1 for 36 h. Immunoprecipitation was performed with anti-HA (**D**) or anti-GFP (**E**) antibodies, followed by Western blotting. (**F**) Confocal microscopy of BHK-21 or ST cells transfected with HA-STUB1 for 24 h and then infected with SVA for 6 or 12 h. Cells were stained with anti-VP1 (green), anti-HA (red), and DAPI (blue). Scale bar, 10 or 20 µm. (**G**) Co-IP analysis of ST cells infected with SVA (MOI = 1) for 12 h, using the anti-VP1 antibody to precipitate endogenous STUB1. (**H**) Confocal microscopy of BHK-21 or ST cells infected with SVA for 6 or 12 h, stained with anti-VP1 (green), anti-STUB1 (red), and DAPI (blue). Scale bar, 10 or 20 µm.

During viral infection, the host leverages the ubiquitin-proteasome system to degrade viral proteins, thereby effectively inhibiting viral replication (18–20). A previous report demonstrated that the SVA VP1 protein is ubiquitinated (32), which consequently raises the question of whether the host can inhibit SVA replication by ubiquitinating VP1 protein. To identify host proteins that interact with VP1, HEK-293T cells were transfected with either a GFP-tagged VP1 plasmid or a control plasmid (GFP-C1). After lysis, the cells were subjected to immunoprecipitation using anti-GFP agarose, followed by liquid chromatography-tandem mass spectrometry (LC-MS) analysis (Table S1). Meanwhile, immunoprecipitates from cells transfected with the control plasmid served as negative controls to account for nonspecific interactions. Additionally, silver staining was performed on the immunoprecipitated proteins to visualize their interactions with GFP-VP1 (Fig. 1A). This screen identified two candidate E3 ubiquitin ligases (Fig. 1B), and STUB1 was selected for further analysis primarily due to its enrichment. Comparative analysis of STUB1 protein sequences revealed significant conservation among porcine, mice, and human orthologs (Fig. 1C). Accordingly, we constructed a plasmid expressing porcine STUB1 for subsequent experiments. To confirm the interaction between the SVA VP1 and STUB1, HEK-293T cells were coexpressed with GFP-C1 or GFP-VP1 and HA-STUB1, followed by forward and reverse co-immunoprecipitation (co-IP) assays using anti-GFP agarose or anti-HA agarose, respectively. Specific bands corresponding to GFP-VP1 and HA-STUB1 were detected (Fig. 1D and E), thus indicating an interaction between these two proteins. Furthermore, confocal imaging revealed colocalization of viral VP1 and HA-STUB1 in SVA-infected BHK-21 and ST cells (Fig. 1F). To further validate the interaction of STUB1 with SVA VP1 during SVA infection, ST cells infected with SVA were harvested at 12 hours post-infection and subjected to immunoprecipitation with an anti-VP1 antibody. The result showed that endogenous STUB1 interacted with VP1 during SVA infection (Fig. 1G). Moreover, confocal microscopy demonstrated colocalization of endogenous STUB1 with VP1 in BHK-21 and ST cells during SVA infection (Fig. 1H). Collectively, these results confirm an interaction between STUB1 and SVA VP1.

### STUB1 negatively regulates SVA replication

**Fig 2:**
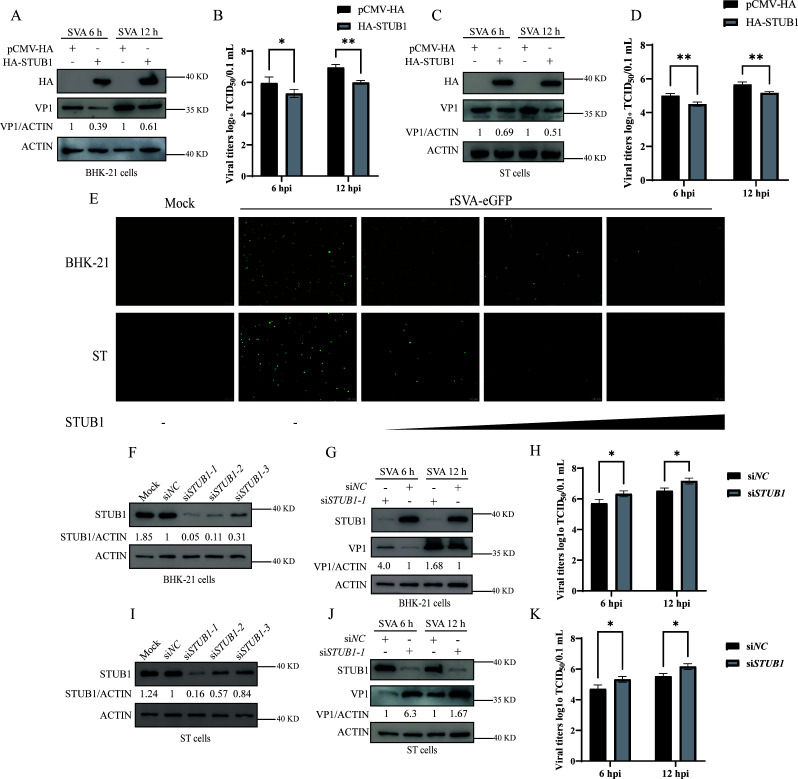
STUB1 negatively regulates SVA replication. (**A–D**) BHK-21 or ST cells transfected with HA-STUB1 or pCMV-HA plasmids were infected with SVA for 6 or 12 h. Extracted proteins and whole-cell culture supernatants were analyzed by Western blotting (**A, C**) and TCID_50_ assay (**B, D**). (**E**) Indirect fluorescence assay (IFA) of BHK-21 or ST cells transfected with increasing concentrations of HA-STUB1 and then infected with rSVV-eGFP (MOI = 1). The number of rSVV-eGFP-positive cells was quantified. (**F, I**) Western blot analysis of STUB1 silencing efficiency in BHK-21 (**F**) or ST cells (**I**) using specific siRNAs (siSTUB1-1/2/3) or nontargeting control (siNC). (**G–H**) BHK-21 cells treated with siSTUB1-1 or siNC were infected with SVA for 6 or 12 h. The extracted proteins and whole-cell culture supernatants were analyzed by Western blotting (**G**) and TCID_50_ assay (**H**). (**J–K**) ST cells treated with siSTUB1-1 or siNC were infected with SVA for 6 or 12 h. The extracted proteins and whole-cell culture supernatants were analyzed by Western blotting (**J**) and TCID_50_ assay (**K**). Data are presented as means ± SD from three independent experiments (ns, not significant; *P < 0.05; **P < 0.01; ***P < 0.001).

To investigate the effect of STUB1 on SVA replication, BHK-21 and ST cells transfected with HA-STUB1 plasmids were infected with SVA for 6 or 12 hours. As shown in Fig. 2A through D, overexpression of STUB1 inhibited SVA replication, as evidenced by reduced viral VP1 expression and lower viral titers at both time points compared to the control group. Additionally, enhanced green fluorescent protein (eGFP)-tagged recombinant SVA (rSVA-eGFP) was used to assess the impact of STUB1 on viral infectivity in BHK-21 and ST cells. The number of rSVA-eGFP-positive cells gradually decreased with increasing HA-STUB1 expression (Fig. 2E). Next, BHK-21 and ST cells were transfected with three distinct siRNAs targeting *STUB1* (si*STUB1*-1, si*STUB1*-2, and si*STUB1*-3). Among these, si*STUB1*-1 was selected for subsequent experiments due to its optimal silencing efficiency in both cell types (Fig. 2F and I). Western blotting and viral titer assays showed that *STUB1* silencing increased VP1 expression and viral titers in BHK-21 and ST cells compared to the control group (Fig. 2G, H, J and K). Taken together, these results demonstrate that STUB1 functions as a negative regulator of SVA replication.

### K177 and K260 in VP1 are necessary for STUB1-mediated, ubiquitination-dependent degradation of VP1

**Fig 3:**
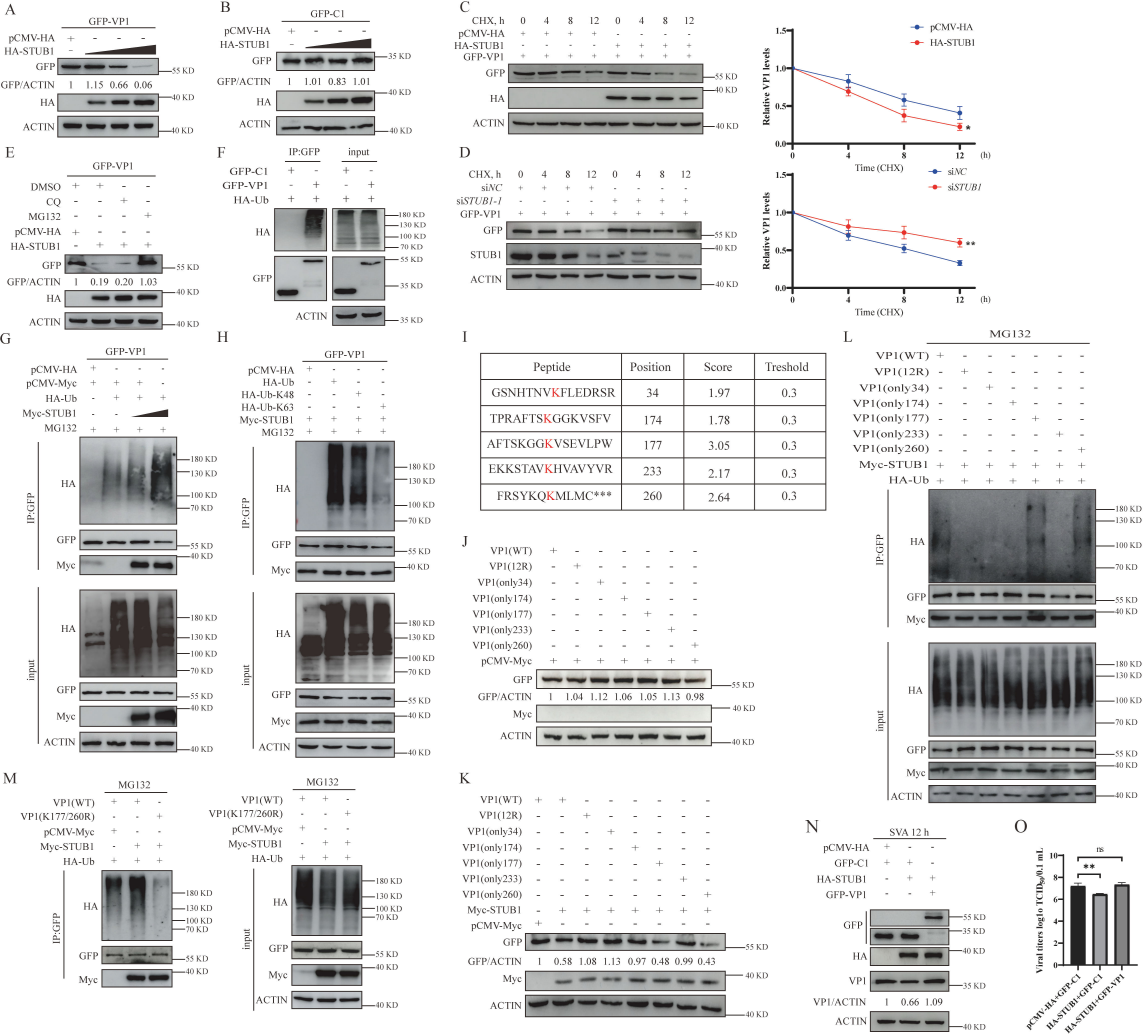
K177 and K260 in VP1 are required for STUB1-mediated ubiquitination degradation of VP1. (**A and B**) Western blot analysis of HEK-293T cells co-transfected with GFP-VP1 (or GFP-C1) and increasing concentrations of HA-STUB1 for 36 h. (**C**) HEK-293T cells co-expressing GFP-VP1 with HA-STUB1 (or pCMV-HA) were treated with cycloheximide (CHX) (100 µg/mL) for the indicated times, followed by Western blotting. (**D**) HEK-293T cells co-expressing GFP-VP1 with siSTUB1-1 (or siNC) were treated with CHX (100 µg/mL) for the indicated time points, followed by Western blotting. (**E**) Western blot analysis of HEK-293T cells co-transfected with HA-STUB1 and GFP-VP1 (or empty vectors) for 24 h and then treated with DMSO, chloroquine (CQ, 20 µM), or MG132 (10 µM) for 12 h. (**F**) HEK-293T cells expressing HA-Ub with GFP-VP1 (or GFP-C1) were analyzed by co-IP and Western blotting. (**G**) HEK-293T cells expressing HA-Ub, GFP-VP1, and increasing concentrations of Myc-STUB1 were treated with MG132 (10 µM) for 12 h, followed by IP with anti-GFP. (**H**) HEK-293T cells expressing GFP-VP1, Myc-STUB1, and HA-Ub (wild-type, K48-only, or K63-only) were treated with MG132 (10 µM) for 12 h, followed by IP with anti-GFP. (**I**) Prediction of potential ubiquitination sites on VP1 using http://www.ubpred.org. (**J and K**) Western blot analysis of HEK-293T cells co-transfected with pCMV-Myc (**J**) or Myc-STUB1 (**K**) and GFP-VP1 (or GFP-tagged VP1 mutants: 12R, K34, K174, K177, K233, or K260) for 36 h. (**L and M**) Ubiquitination assays of HEK-293T cells transfected with the indicated plasmids (GFP-VP1 wild-type or mutants, HA-Ub, and Myc-STUB1) for 24 h, treated with MG132 (10 µM) for 12 h, and analyzed by IP with anti-GFP. (**N and O**) BHK-21 cells transfected with the indicated plasmids were infected with SVA for 12 h, followed by Western blotting (**N**) and TCID₅₀ assay (**O**). Data are presented as means ± SD from three independent experiments (ns, not significant; *P < 0.05; **P < 0.01; ***P < 0.001).

We hypothesized that STUB1 restricts SVA replication by promoting VP1 degradation. To test this, HEK-293T cells were co-transfected with GFP-VP1 or GFP-C1 and varying concentrations of HA-STUB1, and protein extracts were analyzed by Western blotting 36 hours later. The results showed that elevated HA-STUB1 expression caused a dose-dependent decrease in GFP-VP1 levels, with no effect on GFP-C1 (Fig. 3A and B). To further examine the effect of STUB1 on VP1 stability, cycloheximide (CHX) chase assays were performed in HEK-293T cells expressing GFP-VP1, either transfected with HA-STUB1 or STUB1-targeting siRNA (siSTUB1). Western blot analysis revealed that overexpression of HA-STUB1 significantly accelerated GFP-VP1 degradation, whereas STUB1 knockdown markedly stabilized the viral protein (Fig. 3C and D).

STUB1-mediated protein degradation primarily occurs through two major pathways in eukaryotic cells: the ubiquitin-proteasome system and autophagy-lysosome machinery (33). To clarify the mechanism underlying STUB1-mediated VP1 degradation, we evaluated the contribution of these pathways using pharmacological inhibitors. MG132 (a specific proteasome inhibitor) substantially rescued VP1 from degradation, whereas chloroquine (CQ, an autophagy inhibitor) had no protective effect (Fig. 3E), indicating that STUB1 promotes VP1 degradation primarily through the ubiquitin-proteasome system.

Subsequently, ubiquitination of SVA VP1 was confirmed via co-IP and Western blotting, verifying direct ubiquitin-protein conjugation (Fig. 3F). To determine whether STUB1 is involved in VP1 ubiquitination, HEK-293T cells were co-expressed with GFP-VP1, HA-tagged ubiquitin (Ub), pCMV-Myc, or varying concentrations of Myc-STUB1. Co-IP results showed that VP1 ubiquitination increased in a STUB1 concentration-dependent manner (Fig. 3G).

To further elucidate the molecular basis of STUB1-mediated VP1 ubiquitination, HEK-293T cells were co-transfected with GFP-VP1, Myc-STUB1, and either wild-type HA-ubiquitin (HA-Ub) or HA-Ub mutants containing only K48 (HA-Ub-K48) or K63 (HA-Ub-K63) lysine residues. Immunoprecipitation with anti-GFP agarose revealed that VP1 ubiquitination specifically required K48-linked ubiquitin chains, as evidenced by robust ubiquitination signals in the presence of HA-Ub-K48, but not HA-Ub-K63 (Fig. 3H). These results demonstrate that STUB1 specifically promotes K48-linked polyubiquitination of VP1, a modification that typically targets substrates for proteasomal degradation.

To systematically identify potential ubiquitination sites on VP1, we combined bioinformatics prediction with experimental validation. Since ubiquitination predominantly targets lysine residues, we first utilized an online ubiquitination site prediction tool (http://www.ubpred.org) to identify the top five candidate lysine residues in VP1 based on their scores (Fig. 3I). To determine which lysine residues are targeted by STUB1-mediated ubiquitination, we generated a series of GFP-tagged VP1 mutants: a 12R mutant (all lysines mutated to arginines) and five single-lysine mutants retaining only K34, K174, K177, K233, or K260. When co-expressed with HA-STUB1 in HEK-293T cells, only the K177- and K260-retaining mutants exhibited significant degradation (Fig. 3J and K).

For definitive verification, ubiquitination assays were performed by transfecting cells with GFP-VP1 (wild-type or mutants), HA-Ub, and Myc-STUB1, followed by MG132 treatment to accumulate ubiquitinated species. As shown in Fig. 3L, STUB1 specifically mediated ubiquitination of VP1 at K177 and K260, as indicated by robust ubiquitin conjugation to the K177- and K260-retaining mutants. Furthermore, simultaneous mutation of K177 and K260 in VP1 abolished STUB1-mediated ubiquitination, confirming that these sites are specifically targeted by STUB1 (Fig. 3M).

To further demonstrate that STUB1 suppresses SVA replication primarily by degrading VP1, BHK-21 cells were co-transfected with GFP-VP1 (or GFP-C1) and HA-STUB1 and then infected with SVA for 12 hours. Western blotting and TCID₅₀ assays showed that exogenous VP1 expression reversed the STUB1-mediated suppression of SVA yield (Fig. 3N and O). These results indicate that K177 and K260 in VP1 are essential for the STUB1-mediated, ubiquitination-dependent degradation of VP1.

### Heat shock protein 70 (HSP70) or heat shock cognate protein 70 (HSC70) is critical for STUB1-mediated VP1 degradation

**Fig 4:**
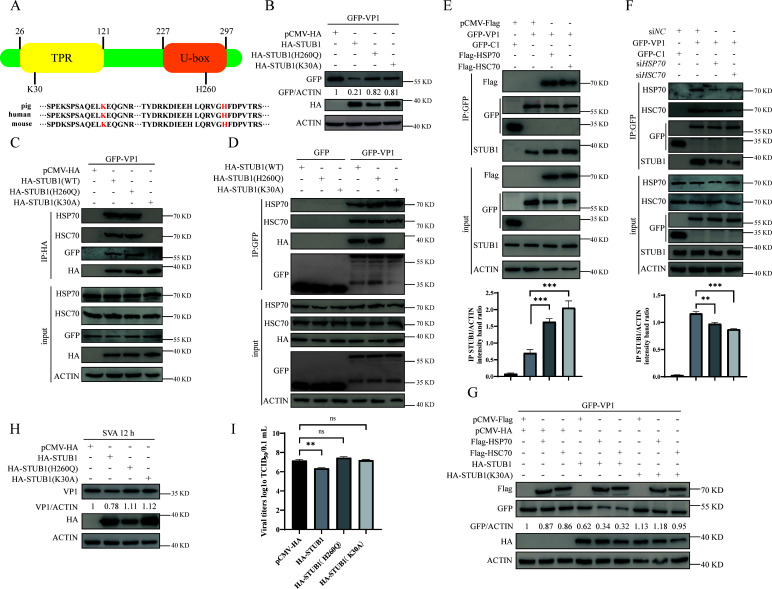
HSP70 and HSC70 facilitate STUB1-mediated degradation of VP1. (**A**) Schematic of STUB1 domains, highlighting K30 (chaperone-binding) and H260 (E3 ligase activity). (**B**) Western blot analysis of HEK-293T cells co-expressing GFP-VP1 with HA-STUB1 (wild-type, K30A, or H260Q) for 36 h. (**C and D**) Co-IP analysis of HEK-293T cells co-expressing GFP-VP1 with HA-STUB1 (wild-type, K30A, or H260Q) for 36 h, using anti-HA (**C**) or anti-GFP (**D**) antibodies. (**E and F**) Co-IP analysis of ST cells transfected with GFP-VP1 and Flag-HSP70/Flag-HSC70 (**E**) or siHSP70/siHSC70 (**F**) for 36 h, using anti-GFP antibody. STUB1 levels were quantified with ImageJ and GraphPad Prism 9.0. (**G**) Western blot analysis of GFP-VP1 expression in HEK-293T cells transfected with the indicated plasmids (Flag-HSP70, Flag-HSC70, HA-STUB1, and HA-STUB1-K30A) for 36 h. (**H and I**) BHK-21 cells transfected with HA-STUB1 (wild-type, K30A, or H260Q) were infected with SVA for 12 h, followed by Western blotting (**H**) and TCID₅₀ assay (**I**). Data are presented as means ± SD from three independent experiments (ns, not significant; *P < 0.05; **P < 0.01; ***P < 0.001).

STUB1-mediated ubiquitination and degradation of substrates often depends on interactions with molecular chaperones (34), typically HSP70 and HSC70 (35). The STUB1-K30A mutant, which harbors a mutation in the tetratricopeptide repeat domain (a chaperone-binding domain), fails to bind chaperones and exhibits reduced substrate interaction (36). Conversely, the STUB1-H260Q mutation in the U-box domain impairs E3 ubiquitin ligase activity, diminishing its ability to ubiquitinate substrates for degradation (36). These two sites are highly conserved in porcine, human, and murine STUB1 (Fig. 4A). To investigate whether these residues are involved in VP1 degradation, HEK-293T cells were co-transfected with GFP-VP1 and either wild-type STUB1 or STUB1 mutants. Compared to wild-type STUB1, both mutants reduced the ability to degrade viral VP1 (Fig. 4B), suggesting that chaperone binding and ubiquitin ligase activity of STUB1 are critical for VP1 degradation.

To explore whether the K30 or H260 mutation affects the STUB1-VP1 interaction, co-IP assays were performed. STUB1-K30A failed to interact with VP1, HSP70, or HSC70, whereas STUB1-H260Q retained interactions with all three (Fig. 4C). This indicates that the STUB1-K30A mutation abrogates chaperone binding, which may underlie its inability to interact with VP1, highlighting the essential role of STUB1’s chaperone-binding activity in mediating VP1 interaction.

Reciprocal co-IP assays using anti-GFP agarose further revealed that while the STUB1-K30A mutation disrupted STUB1-VP1 binding, it did not affect interactions between VP1 and HSP70 or HSC70 (Fig. 4D). This supports a model in which heat shock proteins act as molecular adapters, bridging STUB1 and SVA VP1.

To examine the role of HSP70 and HSC70 in the STUB1-VP1 interaction, ST cells cotransfected with GFP-VP1 and Flag-HSP70 or Flag-HSC70 were lysed 36 hours and subjected to immunoprecipitation with an anti-GFP antibody. VP1 exhibited a stronger interaction with STUB1 when HSP70 or HSC70 was overexpressed (Fig. 4E), whereas this interaction was weakened when HSP70 or HSC70 was knocked down using siRNA (Fig. 4F).

To further define the role of these chaperones in STUB1-mediated VP1 degradation, HEK-293T cells were co-transfected with Flag-HSP70, Flag-HSC70, HA-STUB1, HA-STUB1-K30A, and GFP-VP1. Both HSP70 and HSC70 significantly enhanced STUB1-mediated VP1 degradation, whereas they had no effect when combined with STUB1-K30A (Fig. 4G). Importantly, disruption of either chaperone-binding (K30) or catalytic (H260) sites abolished STUB1’s antiviral activity, as evidenced by increased VP1 expression and viral titers (Fig. 4H and I). Collectively, these results indicate that HSP70 and HSC70 play critical roles in STUB1-mediated VP1 degradation.

### SVA infection downregulates STUB1 expression.

**Fig 5:**
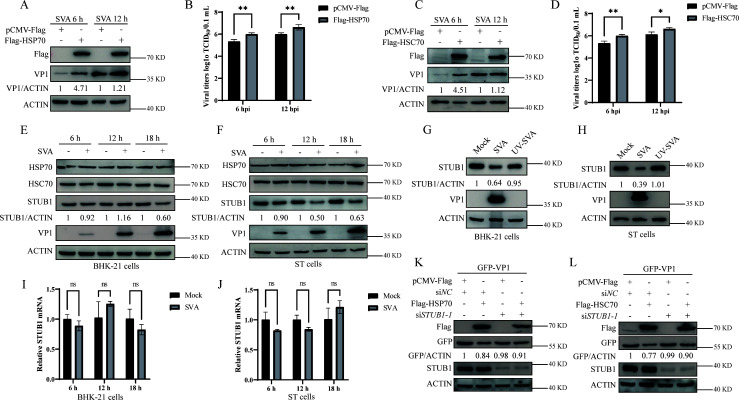
SVA infection downregulates STUB1 expression. (**A and B**) BHK-21 cells transfected with Flag-HSP70 (or pCMV-Flag) were infected with SVA for 6 or 12 h, followed by Western blotting (**A**) and TCID₅₀ assay (**B**). (**C and D**) BHK-21 cells transfected with Flag-HSC70 (or pCMV-Flag) were infected with SVA for 6 or 12 h, followed by Western blotting (**C**) and TCID₅₀ assay (**D**). (**E and F**) Western blot analysis of STUB1, HSP70, and HSC70 in BHK-21 (**E**) or ST cells (**F**) mock-infected (PBS) or infected with SVA (MOI = 1) for 6, 12, or 18 h. (**G and H**) Western blot analysis of STUB1 in BHK-21 (**G**) or ST cells (**H**) infected with SVA or UV-inactivated SVA for 18 h. (**I and J**) STUB1 mRNA levels in BHK-21 (**I**) or ST cells (**J**) mock-infected or infected with SVA for 6, 12, or 18 h (MOI = 1), analyzed by RT-qPCR (normalized to GAPDH). (**K and L**) Co-IP analysis of BHK-21 cells transfected with the indicated plasmids or siRNAs for 36 h, using specific antibodies. Data are presented as means ± SD from three independent experiments (ns, not significant; *P < 0.05; **P < 0.01; ***P < 0.001).

To investigate the regulatory role of HSP70 and HSC70 in SVA replication, cells overexpressing these chaperones were infected with SVA for 6 or 12 hours. Despite their role in enhancing STUB1-mediated VP1 degradation (Fig. 4), both HSP70 and HSC70 promoted viral replication (Fig. 5A through D), consistent with our previous report (37). This paradox prompted us to investigate the underlying mechanism.

STUB1 expression was downregulated in SVA-infected BHK-21 and ST cells, whereas HSP70 and HSC70 levels remained unchanged (Fig. 5E and F). This reduction in STUB1 was dependent on active SVA replication (Fig. 5G and H), with no change in STUB1 mRNA levels (Fig. 5I and J), suggesting post-translational regulation of STUB1.

To determine whether STUB1 is required for HSP70-/HSC70-mediated VP1 degradation, STUB1 was silenced in cells expressing HSP70 or HSC70. STUB1 silencing attenuated VP1 degradation induced by HSP70 or HSC70 (Fig. 5K and L), indicating that STUB1 is necessary for chaperone-mediated VP1 degradation. Taken together, these results suggested that SVA infection downregulates STUB1 expression to disrupt HSP70-/HSC70-mediated VP1 degradation.

### SVA 3C protein antagonizes STUB1-mediated VP1 degradation by reducing STUB1 expression.

**Fig 6:**
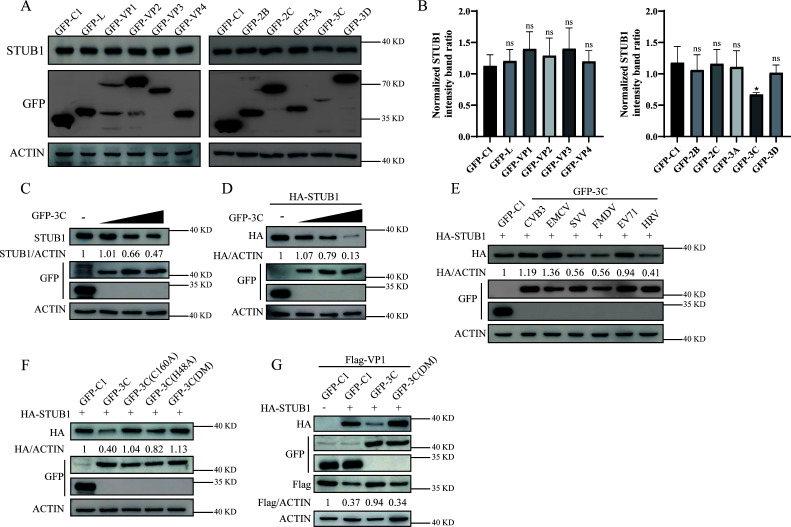
SVA 3Cpro antagonizes STUB1-mediated VP1 degradation by reducing STUB1 expression. (**A**) BHK-21 cells transfected with plasmids encoding viral proteins for 24 h were analyzed by Western blotting. β-actin served as a loading control. (**B**) Quantification of relative STUB1 levels in (**A**) using ImageJ and GraphPad Prism 9.0. (**C**) Dose-dependent effect of GFP-3C on endogenous STUB1: BHK-21 cells transfected with increasing amounts of GFP-3C for 24 h were analyzed by Western blotting. (**D**) Dose-dependent effect of GFP-3C on HA-STUB1: HEK-293T cells transfected with GFP-3C and HA-STUB1 for 24 h were analyzed by Western blotting. (**E**) Specificity of 3Cpro from picornaviruses: HEK-293T cells transfected with GFP-3C (SVA, CVB3, EMCV, FMDV, EV71, or HRV) and HA-STUB1 were analyzed by Western blotting. (**F**) Requirement for catalytic activity: HEK-293T cells transfected with HA-STUB1 and GFP-3C (wild-type or catalytic mutants: H48A, C160A, and DM) were analyzed by Western blotting. (**G**) Effect of 3Cpro on STUB1-mediated VP1 degradation: HEK-293T cells co-transfected with HA-STUB1, Flag-VP1, and GFP-3C (wild-type or DM) were analyzed by Western blotting. Data are presented as means ± SD from three independent experiments (ns, not significant; *P < 0.05; **P < 0.01; ***P < 0.001).

To identify the SVA protein responsible for STUB1 downregulation, BHK-21 cells were transfected with various viral proteins (13). Only SVA 3C protease (3Cpro) induced STUB1 degradation, mimicking the pattern observed during SVA infection (Fig. 6A and B). Moreover, increased GFP-3C expression caused a dose-dependent decrease in both endogenous STUB1 and HA-STUB1 levels (Fig. 6C and D).

We also examined the effect of 3Cpro from other picornaviruses, including coxsackievirus B3 (CVB3), encephalomyocarditis virus (EMCV), foot and mouth disease virus (FMDV), enterovirus 71 (EV71), and human rhinovirus (HRV). SVV, FMDV, and HRV 3Cpro decreased STUB1 expression, whereas CVB3, EMCV, and EV71 3Cpro had no effect (Fig. 6E).

SVA 3Cpro typically degrades host proteins directly via its protease activity (38). To investigate this, we co-expressed HA-STUB1 with GFP-3C or GFP-3C mutants harboring mutations in critical protease residues (GFP-3C-H48A, GFP-3C-C160A, and GFP-3C-DM [H48A-C160A]) (39). Mutational analysis showed that catalytically inactive 3Cpro (H48A/C160A) failed to promote STUB1 degradation (Fig. 6F), demonstrating that protease activity is essential.

To assess the functional consequences, we examined 3Cpro’s impact on STUB1-mediated antiviral activity. Co-expression experiments in HEK-293T cells showed that wild-type 3Cpro, but not the protease-deficient mutant (3Cpro-DM), effectively counteracted STUB1-mediated VP1 degradation via specific reducing STUB1 protein levels (Fig. 6G).

### Replication capacity of SVA mutants is enhanced *in vitro* and *in vivo.*

**Fig 7:**
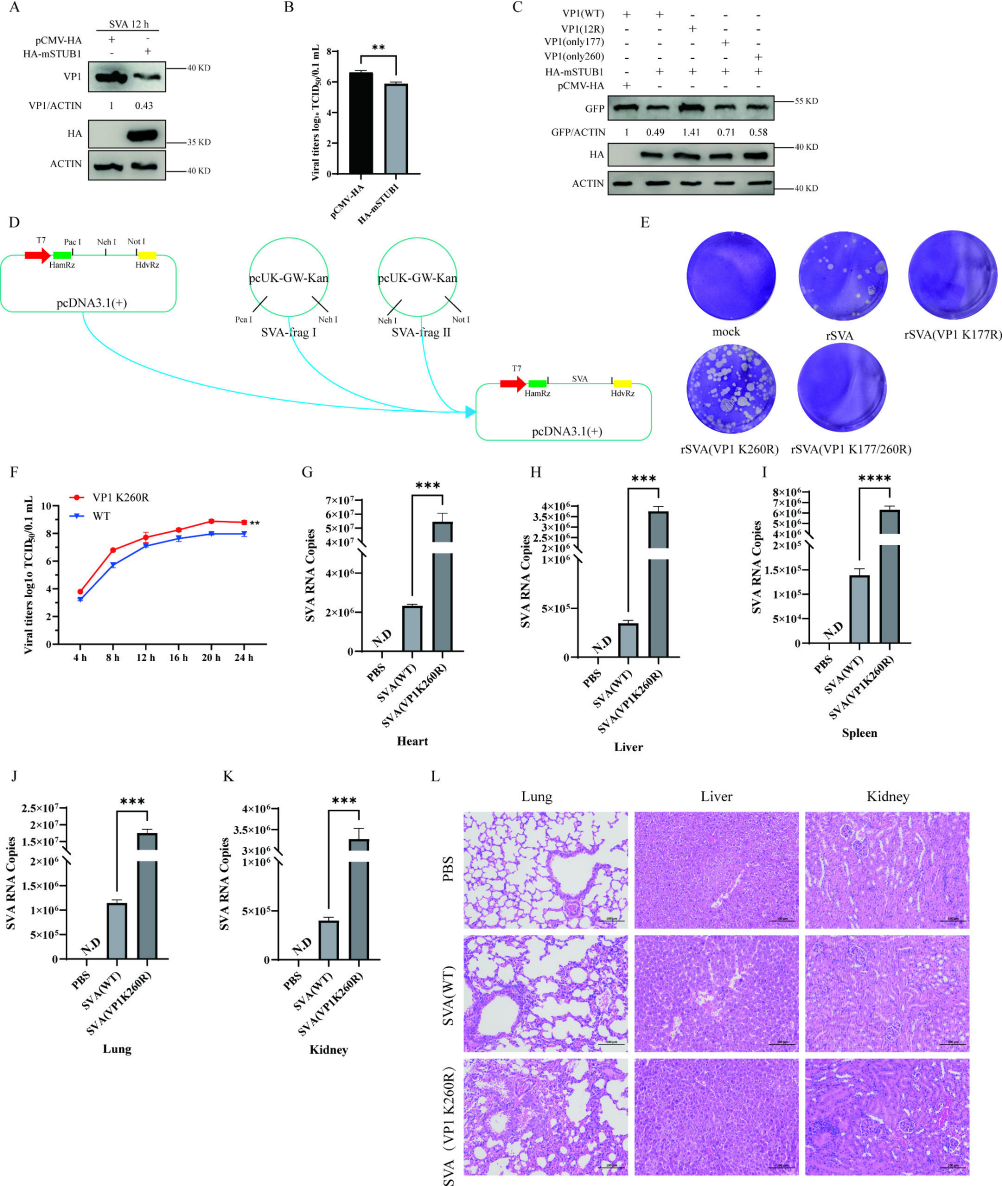
Enhanced replication and pathogenicity of SVA VP1-K260R mutant *in vitro* and *in vivo*. (**A and B**) Mouse STUB1 inhibits SVA replication: BHK-21 cells transfected with HA-mSTUB1 (mouse) or pCMV-HA were infected with SVA for 12 h, analyzed by Western blotting (**A**) and TCID₅₀ assay (**B**). (**C**) Mouse STUB1 degrades VP1 mutants: HEK-293T cells co-transfected with HA-mSTUB1 and GFP-VP1 (wild-type, K177-only, or K260-only) were analyzed by Western blotting. (**D**) Schematic of infectious SVA cDNA clone construction for wild-type and VP1 mutants (K177R, K260R, and double mutant). (**E**) Plaque morphology of wild-type rSVA and rSVA-VP1-K260R in BHK-21 cells (K177R mutant was nonviable). (**F**) Growth curve of rSVA and rSVA-VP1-K260R in BHK-21 cells. (**G–K**) Viral loads in tissues of BALB/c mice infected with rSVA or rSVA-VP1-K260R (5 days post-infection), quantified by RT-qPCR. (**L**) Histopathological analysis of the lung, liver, and kidney from infected mice (hematoxylin and eosin staining). Data are presented as means ± SD (N.D., not detected; ***P < 0.001).

Given the high sequence homology between murine and porcine STUB1 proteins, we investigated the effect of murine STUB1 on SVA replication. Murine STUB1 also inhibited SVA replication (Fig. 7A and B) and degraded VP1 containing only 177 or 260 (Fig. 7C). To evaluate the role of VP1 K177 and K260 in SVA replication, we engineered viruses harboring K177R, K260R, or double mutant (Fig. 7D). The number of plaques formed by rSVA (VP1 K260R) exceeded that of wild-type rSVA, indicating higher viral titers (Fig. 7E). In contrast, no plaques were detected for rSVA (VP1 K177R), suggesting that K177 mutation is lethal to SVA. Additionally, rSVA (VP1 K260R) exhibited significantly higher titers than the wild-type rSVA (Fig. 7F).

To further characterize the functional role of K260, we used a mouse model of SVA infection established via oral administration (11). At 5 days post-infection (dpi), rSVA (VP1 K260R) showed higher viral loads in the heart, liver, spleen, kidney, and lung tissues compared to wild-type rSVA (Fig. 7G through K). Notably, lung tissue from mice infected with rSVA (VP1 K260R) exhibited more severe damage (thickened alveolar walls with focal granulocyte infiltration and compensatory alveolar dilation with widened interalveolar septa) than from wild-type rSVA-infected mice, with no significant pathology in the liver or kidney (Fig. 7L). Taken together, these findings indicate that rSVA (VP1 K260R) has enhanced replication capacity and pathogenicity *in vitro* and *in vivo*.

## DISCUSSION

Senecavirus A (SVA), an emerging virus associated with porcine idiopathic vesicular disease, causes significant global economic losses (2–4, 40). The SVA VP1 protein is critical for viral attachment and entry, making it a promising target for anti-SVA strategies (14). For example, the selective autophagy receptor SQSTM1/p62 inhibits SVA replication by targeting VP1 (32), and OPTN suppresses SVA replication by promoting VP1 degradation (41). Components of the ubiquitin-proteasome system, particularly E3 ligases, often act as intrinsic antiviral factors (42). Here, we demonstrate that the E3 ubiquitin ligase STUB1 binds to and degrades the SVA VP1, with VP1 K177 and K260 being essential for STUB1-mediated, ubiquitination-dependent degradation.

Previous studies on STUB1 in viral replication have focused on regulating immune signaling pathways. For instance, mixed-lineage leukemia 5 inhibits the antiviral innate immune response by facilitating STUB1-mediated degradation of retinoic acid-inducible gene I (43); receptor for activated C kinase 1 enhances bovine ephemeral fever virus replication by upregulating STUB1 expression, which degrades mitochondrial antiviral signaling proteins (MAVS) (44); and cytoplasmic signal transducer and activator of transcription 4 promotes antiviral type I interferon (IFN) production by inhibiting STUB1-mediated degradation of retinoic acid-inducible gene I (45). These findings highlight STUB1’s complex roles in viral infections via immune modulation. In contrast, here we identify STUB1 as a key host restriction factor that negatively regulates SVA replication by targeting VP1 for ubiquitination and degradation, consistent with reports that STUB1 mediates degradation of the HIV-1 Tat protein (46).

STUB1 is an E3 ubiquitin ligase that ubiquitinates and degrades substrates (21), and our results confirm that STUB1-mediated VP1 degradation occurs primarily via the ubiquitin-proteasome degradation pathway. Ubiquitin contains seven lysine residues (K6, K11, K27, K29, K33, K48, and K63) that form chains on target proteins (47, 48). K48-linked ubiquitin chains predominantly target proteins for proteasomal degradation, whereas K63-linked chains are involved in processes such as DNA damage repair, protein stabilization, and kinase activation (49, 50). We show that STUB1 overexpression increases VP1 ubiquitination in a dose-dependent manner, specifically inducing K48-linked (but not K63-linked) ubiquitination, consistent with proteasomal targeting.

Identifying specific ubiquitination sites on target proteins helps elucidate the relationship between the ubiquitin-proteasome system and protein function (51). For example, dengue virus nonstructural protein 3 (NS3) is ubiquitinated at K104 by TRIM69 (52), and STING is modified with K63-linked chains at K150 by TRIM56 (53). SVA VP1 contains 12 lysine residues, with K177 and K260 identified as STUB1 targets.

Molecular chaperones, particularly HSP70 and HSC70, are known to participate in STUB1-mediated protein degradation (54, 55). STUB1 residues K30 and H260 are crucial for chaperone binding and ubiquitination activity, respectively (36). We explored the effects of HSP70, HSC70, and these residues on VP1 degradation and the STUB1-VP1 interaction. Consistent with previous reports (56, 57), STUB1 mutants at these residues failed to degrade VP1, and the K30A mutant disrupted interactions with VP1 or chaperones but not between VP1 and HSP70/HSC70. This suggests that STUB1 does not directly interact with VP1 but instead requires HSP70 or HSC70 as intermediaries. Furthermore, we found that HSP70 and HSC70 promote STUB1-mediated VP1 degradation by enhancing their interaction.

During evolution, viruses have evolved to exploit host proteins that facilitate replication while degrading those that hinder survival. Here, HSP70 and HSC70 enhance the STUB1-VP1 interaction to promote VP1 degradation, suggesting that they inhibit SVA replication. This contradicts our previous finding that HSP70 positively regulates SVA replication by stabilizing viral L and 3D proteins (37). This paradox implies a mechanism that inhibits STUB1-mediated VP1 degradation in the presence of HSP70/HSC70 during SVA infection. STUB1 expression is downregulated in SVA-infected cells, whereas HSP70/HSC70 levels remain unchanged. Thus, SVA-mediated STUB1 downregulation may shift HSP70/HSC70 function toward stabilizing viral nonstructural proteins rather than degrading VP1, thereby promoting replication.

SVA 3Cpro typically cleaves or degrades host proteins via its protease activity to evade antiviral mechanisms. For example, it mediates nucleolin cleavage and redistribution (39), disrupts mitochondrial DNA-mediated immune sensing (58), and cleaves hnRNPK (59) to facilitate replication. Our results show that catalytically inactive 3Cpro loses its ability to degrade STUB1 and antagonizes STUB1-mediated VP1 degradation, indicating that protease activity is critical for counteracting host antiviral responses. However, like previous studies (16, 38), we did not detect cleaved fragments of STUB1, possibly due to rapid degradation of small fragments; the mechanism of 3Cpro-mediated STUB1 degradation requires further investigation.

Reverse genetics is pivotal for validating the functional roles of specific amino acid residues in viral proteins. For example, recombinant FMDV with the VP1-K200R mutation abrogates RNF5-mediated antiviral suppression (60), and recombinant Tembusu virus with NS1-K141R shows enhanced replication in avian hosts (61). We engineered VP1 mutants to explore the roles of K177 and K260 in SVA replication. The K177R mutation was lethal, whereas K260R enhanced replication. Mice infected with rSVA (VP1 K260R) showed higher viral loads in multiple tissues and more severe lung damage than those infected with wild-type virus. Consistent with the findings of Li et al. (62), the highest viral loads were observed in the heart, likely due to the close genomic relationship between SVA and Cardioviruses.

**Fig 8:**
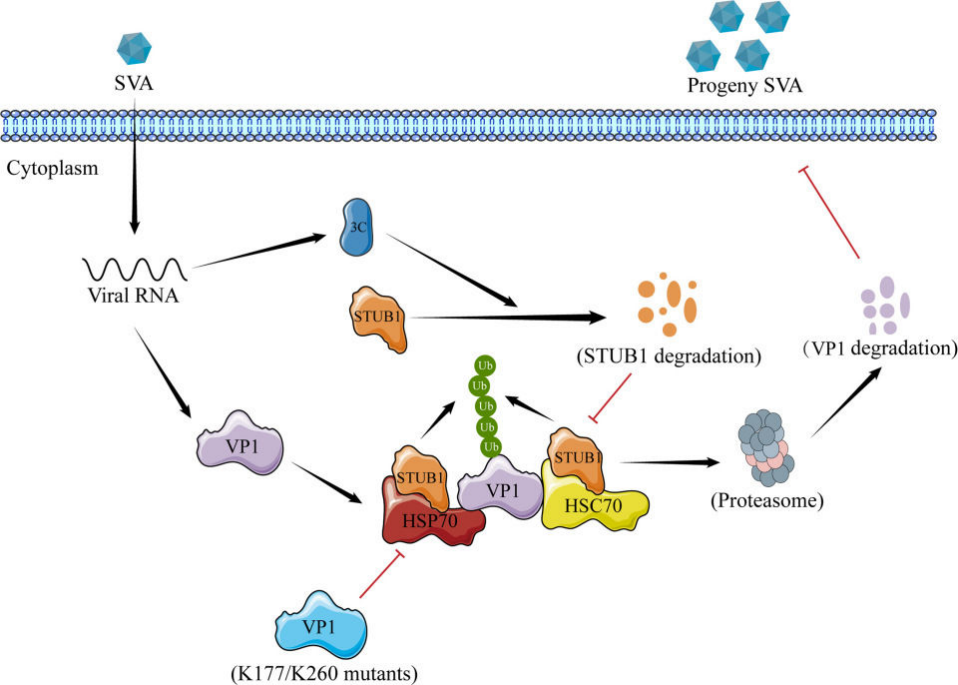
Schematic model of STUB1-mediated suppression of SVA replication. STUB1 interacts with SVA VP1 and promotes its degradation via K48-linked ubiquitination at K177 and K260, thereby inhibiting viral replication. HSP70 and HSC70 enhance this process by strengthening the STUB1-VP1 interaction. During infection, SVA 3Cpro degrades STUB1 (via its protease activity), blocking VP1 degradation and antagonizing the host antiviral response.

In conclusion, our results demonstrate that STUB1 inhibits SVA replication by promoting VP1 degradation, while SVA 3Cpro antagonizes this process by reducing STUB1 expression (Fig. 8). Understanding STUB1 regulatory mechanisms provides insights into strategies for preventing and controlling SVA infections.

## MATERIALS AND METHODS

### Cells, viruses, and reagents

BHK-21, ST, and HEK-293T cells were obtained from the American Type Culture Collection (ATCC) and cultured in Dulbecco’s modiﬁed Eagle’s medium (DMEM; Gibco, Waltham, MA, USA) supplemented with 5%–10% fetal bovine serum (FBS; Gibco), streptomycin, and penicillin at 37°C in a 5% CO_2_ incubator. SVA CHhb17 strain and an anti-SVA VP1 monoclonal antibody were preserved in our laboratory. The eGFP-tagged recombinant SVA (rSVA-eGFP) was kindly provided by Dr. Fuxiao Liu (Qingdao Agricultural University). Commercially sourced antibodies included the following: rabbit anti-GFP (D110008-0200; Sangon, Shanghai, China), mouse anti-β-actin (D191047; Sangon), rabbit anti-STUB1 (A11751; ABclonal), rabbit anti-HSP70 (A20819; ABclonal, Woburn, MA, USA), rabbit anti-HSC70 (A0415; ABclonal), mouse anti-Flag (F4049; Sigma, St. Louis, MO, USA), mouse anti-Myc (05-419;Merck, St. Louis, MO, USA), mouse anti-HA (H3663; Sigma), horseradish peroxidase (HRP)-conjugated anti-rabbit and -mouse secondary (A0545 or A9044; Sigma), and tetramethylrhodamine isothiocyanate (TRITC)-conjugated rabbit anti-mouse antibodies (ab6799; Abcam, Cambridge, UK).

### Plasmid construction and transfection

Plasmids encoding the SVA *VP1* gene were preserved in our laboratory (13). *STUB1*, *HSP70*, and *HSC70* genes derived from PK-15 cells or RAW cells were cloned into pCMV-HA, pCMV-Flag, or pCMV-Myc vectors. All primers used for plasmid construction are listed in Table 1. For transfection, BHK-21, ST, or HEK-293T cells were cultured to 70%–80% confluence and transfected with the indicated plasmids using Lipofectamine 2000 (11668019; Invitrogen, Carlsbad, CA, USA), following the manufacturer’s protocols.

**TABLE 1:** Primers and siRNA in this study

Primers and siRNA	Sequence (5′−3′)
HA-STUB1-F	TTGAATTCGGATGAAGGGCAAGGAG
HA-STUB1-R	TTGGTACCTCAGTAGTCCTCCACCCAGC
Myc-STUB1-F	TTGAATTCGGATGAAGGGCAAGGAG
Myc-STUB1-R	TTGGTACCTCAGTAGTCCTCCACCCAGC
HA-mSTUB1-F	TTGAATTCGGATGAAGGGCAAGGAGGAAAAGG
HA-mSTUB1-R	TTGGTACCTCAATAGTCCTCTACCCAGCC
Flag-HSP70-F	TTGTCGACTATGTCCGCTGCAAGAGAAGTGG
Flag-HSP70-R	TTGGTACCTTAATCAACCTCCTCAATGACAGGGC
Flag-HSC70-F	TTGTCGACTATGTCTAAGGGACCTGCAGTTGG
Flag-HSC70-R	TTGGTACCTTAGTCAACCTCCTCAATGGTGGGC
Flag-VP1-F	TTAAGCTTATGTCCACCGACAACGCCGAG
Flag-VP1-R	TTGGTACCTTATTGCATCAGCATCTTTTGCTTGTAGC
siHSP70	AGGCCAACAAGAUCACCAUTT
siSTUB1-1	GCCAGUCUGUGAAAGCACATT
siSTUB1-2	GCAGACAUGGAUGAGCUCUTT
siSTUB1-3	GCCAUGAAGGAGGUCAUUGTT
siNC	UUCUCCGAACGUGUCACGUTT

### Silver staining and mass spectrometric identiﬁcation of proteins

HEK-293T cells were transfected with plasmids expressing GFP-VP1 or GFP (control) and lysed 36 h post-transfection in NP 40 buffer (10 mM Tris pH 7.5, 150 mM NaCl, 0.5% NP-40, 0.5 mM EDTA). Lysates were incubated with anti-GFP mAb-conjugated beads (GAN-50-1000; Lablead, Beijing, China) for immunoprecipitation. Immunoprecipitated proteins were washed three times with washing buffers (10 mM Tris pH 7.5, 150 mM NaCl, 0.05% NP-40, 0.5 mM EDTA), separated by sodium dodecyl sulfate-polyacrylamide gel electrophoresis (SDS-PAGE), and visualized using a silver staining kit (#24612; Thermo Fisher Scientiﬁc) according to the manufacturer’s instructions. Differential protein bands GFP-VP1 and GFP control lanes were manually excised for mass spectrometric analysis.

### Viral infection and 50% tissue culture infectious dose (TCID_50_) assay

BHK-21 or ST cells (transfected or untransfected with plasmid) were washed with phosphate-buffered saline (PBS) and infected with SVA at a multiplicity of infection (MOI) of ^1^ for 1 h at 37°C. After removing unbound virus, cell culture supernatants were collected at specified time points, and viral titers were determined. Monolayer BHK-21 or ST cells in 96-well plates were inoculated with 100 μL of 10-fold serial dilutions of samples (eight replicates per dilution) and cultured until cytopathic effects (CPE) were observed. The 50% tissue culture infectious dose (TCID_50_) was determined using the Spearman and Karber’s method.

### siRNA transfection (RNAi)

siRNAs targeting STUB1 and HSP70 genes were designed and synthesized by GenePharma. The siRNA targeting HSC70 (siHSC70, sc-29349) was purchased from Santa Cruz. Cells were transfected with siRNA using Lipofectamine RNAiMAX (13778; Invitrogen) for 36 h, followed by SVA infection or plasmid transfection. Samples were then analyzed by Western blotting and TCID_50_ assay. Sequences of siRNAs are listed in Table 1.

### RNA extraction and reverse transcription-quantitative polymerase chain reaction (RT-qPCR)

Total RNA was extracted from cells using TRIzol reagent (15596018; Invitrogen) and reverse-transcribed into cDNA using HiScript III RT SuperMix (R323-01; Vazyme, China). Quantitative PCR was performed using Taq Pro Universal SYBR qPCR Master Mix (Q712-02; Vazyme) on a LightCycler 96 system (Roche, Basel, Switzerland), with three technical replicates per sample. Relative mRNA levels of the *STUB1* gene were calculated using the compare active cycle threshold (2^−ΔΔCT^) method and normalized to glyceraldehyde-3-phosphate dehydrogenase (GAPDH) mRNA. SVA RNA copy numbers were quantified using a standard curve (Y = -3.3814X + 36.59) generated from conserved sequences of the SVA *3D* gene. Primer sequences are listed below: porcine GAPDH: F (TCG GAGTGAACGGATTTGGC) and R (TGACAAGCTTCCC-GTTCTCC), porcine STUB1: F (GGAGAACGAGCTGCACTCTT) and R (TGGTTCCGCTGACACTCTTC), hamster GAPDH: F GTCATC ATCTCCGCCCCTTC and R CCGTGGTCATGAGTCCTTCC, and hamster STUB1: F (TGCCCTTCGCATTGCTAAGA) and R TCCTCCAGTTCCCTCTCTCG, and SVA-3D: F (CCAACAAGGGTTCCGTCTTC) and R (TTGGACGAATTTGCGTTTTAGA).

### Cycloheximide (CHX) chase assay

Cells co-transfected with GFP-VP1, HA-STUB1, or si*STUB1-1* were treated with CHX (100 μg/mL) for various durations to block *de novo* protein synthesis. Proteins were extracted and analyzed by Western blotting.

### Immunoﬂuorescence assays and confocal microscopy

BHK-21 or ST cells (80%–90% confluence in 24-well plates) were transfected with the indicated plasmids for 12 h (with or without rSVA-eGFP infection). Cells were fixed with 4% paraformaldehyde, washed, and incubated with primary antibodies, followed by TRITC-conjugated secondary antibodies and DAPI (nuclear stain). Fluorescent images were captured using an immunoﬂuorescence microscope (IX73; Olympus, Tokyo, Japan) or confocal microscope (TCS SP8 STED; Leica, Wetzlar, Germany).

### Co-immunoprecipitation and western blotting

HEK-293T or ST cells were co-transfected with the indicated plasmids for 36 h, lysed with NP40 buffer containing phenylmethanesulfonyl fluoride (PMSF) (ST506; Beyotime), and immunoprecipitated with anti-GFP agarose (PGA025; Lablead) or anti-HA agarose (HNA-50-1000; Lablead) at 4°C for 1 h with rotation. For endogenous co-IP, the mouse anti-VP1 monoclonal antibody was conjugated to Protein A/G Plus Agarose overnight at 4°C and then incubated with lysates of SVA-infected ST cells for 12 h at 4°C. Immunoprecipitates or total cell lysates were separated by SDS-PAGE, transferred to nitrocellulose membranes (66485; Pall, Port Washington, NY, USA), blocked with 5% non-fat milk, and probed with primary and secondary antibodies. Signals were detected using an AMERSHAM ImageQuant800 chemiluminescence imaging system (GE, Chicago, IL, USA).

### Plaque assays

BHK-21 cells were seeded in 6-well plates and grown to 100% confluence. Cells were inoculated with 100 TCID_50_ of virus and incubated for 1 h at 37°C and then overlaid with DMEM containing 2% heat-inactivated FBS and 1% low-melting-point agarose. After 24 h, plaques were visualized by staining with 1% crystal violet in methanol for 5 h at 37°C.

### Construction of infectious SVA cDNA clones

Based on the SVA CHhb17 genome (MG983756), two fragments were synthesized: SVA Frag I (positions 1–3,514, flanked by PacI and NheI) and SVA Frag II (positions 3,515–7,280, flanked by NheI and NotI). These were cloned into pUC-GW-Kan and then ligated subsequently into pcDNA-rSVAuni (a modified pcDNA3.1(+) vector containing a CMV promoter, T7 promoter, SVA hammerhead ribozyme [HamRz], restriction sites, hepatitis delta virus ribozyme [HdvRz], and T7 terminator) using double enzyme digestion. Plasmids pUC-GW-Kan and pcDNA-rSVAuni were kindly provided by Dr. Shichong Han (International Joint Research Center of National Animal Immunology, College of Veterinary Medicine, Henan Agricultural University). To rescue viruses, full-length cDNA plasmids were transfected into BHK-21 cells using Lipofectamine 2000. When ~90% CPE was observed, supernatants were collected and serially passaged five times in BHK-21 cells. Virus stocks were stored at −80°C.

### Animal experiments

Fifteen 3-week-old female BALB/c mice (Yangzhou University Laboratory Animal Center) were housed in pathogen-free facilities and randomly divided into three groups (n = 5 each): negative control, rSVA-infected, and rSVA (VP1K260R)- infected. Mice were infected with 2 × 10^7^ TCID_50_ of virus via oral administration, as described previously (11). At 5 days post-infection (dpi), mice were euthanized, and tissues (heart, liver, spleen, kidneys, and lungs) were collected. Tissues were either processed for RT-qPCR (viral load quantification) or fixed in 4% paraformaldehyde, embedded in paraffin, sectioned (4 μm), and stained with hematoxylin and eosin (HE) for histopathological analysis.

### Statistical analysis

Statistical differences were evaluated using one-way analysis of variance (ANOVA) or Student’s *t*-test with GraphPad Prism 9.0 software (GraphPad Software, Boston, USA). P<0.05 was considered statistically significant.

## Data Availability

All relevant data are in the manuscript and its supplemental material.

## SUPPLEMENTAL MATERIAL

Table S1 jvi.01152-25-s0001.xlsx Proteins found to bind to SVA VP1 using liquid chromatography-tandem mass spectrometry.

ASM does not own the copyrights to Supplemental Material that may be linked to, or accessed through, an article. The authors have granted ASM a non-exclusive, world-wide license to publish the Supplemental Material files. Please contact the corresponding author directly for reuse.

## References

[1] Hales LM, Knowles NJ, Reddy PS, Xu L, Hay C, Hallenbeck PL. 2008. Complete genome sequence analysis of Seneca Valley virus-001, a novel oncolytic picornavirus. J Gen Virol 89:1265–1275. doi:10.1099/vir.0.83570-0 18420805

[2] Leme RA, Zotti E, Alcântara BK, Oliveira MV, Freitas LA, Alfieri AF, Alfieri AA. 2015. Senecavirus A: an emerging vesicular infection in Brazilian Pig Herds. Transbound Emerg Dis 62:603–611. doi:10.1111/tbed.12430 26398783

[3] Saeng-Chuto K, Rodtian P, Temeeyasen G, Wegner M, Nilubol D. 2018. The first detection of Senecavirus A in pigs in Thailand, 2016. Transbound Emerg Dis 65:285–288. doi:10.1111/tbed.12654 28474854

[4] Sun D, Vannucci F, Knutson TP, Corzo C, Marthaler DG. 2017. Emergence and whole-genome sequence of Senecavirus A in Colombia. Transbound Emerg Dis 64:1346–1349. doi:10.1111/tbed.12669 28714178

[5] Qian S, Fan W, Qian P, Chen H, Li X. 2016. Isolation and full-genome sequencing of Seneca Valley virus in piglets from China, 2016. Virol J 13:173. doi:10.1186/s12985-016-0631-2 27756396 PMC5069920

[6] Zhu Z, Yang F, Chen P, Liu H, Cao W, Zhang K, Liu X, Zheng H. 2017. Emergence of novel Seneca Valley virus strains in China, 2017. Transbound Emerg Dis 64:1024–1029. doi:10.1111/tbed.12662 28544501

[7] Miles LA, Burga LN, Gardner EE, Bostina M, Poirier JT, Rudin CM. 2017. Anthrax toxin receptor 1 is the cellular receptor for Seneca Valley virus. J Clin Invest 127:2957–2967. doi:10.1172/JCI93472 28650343 PMC5531414

[8] Kennedy EM, Denslow A, Hewett J, Kong L, De Almeida A, Bryant JD, Lee JS, Jacques J, Feau S, Hayes M, McMichael EL, Wambua D, Farkaly T, Rahmeh AA, Herschelman L, Douglas D, Spinale J, Adhikari S, Deterling J, Scott M, Haines BB, Finer MH, Ashburn TT, Quéva C, Lerner L. 2022. Development of intravenously administered synthetic RNA virus immunotherapy for the treatment of cancer. Nat Commun 13:5907. doi:10.1038/s41467-022-33599-w 36207308 PMC9546900

[9] Liu F, Huang Y, Wang Q, Shan H. 2020. Construction of eGFP-tagged Senecavirus A for facilitating virus neutralization test and antiviral assay. Viruses 12:283. doi:10.3390/v12030283 32150804 PMC7150990

[10] Joshi LR, Mohr KA, Clement T, Hain KS, Myers B, Yaros J, Nelson EA, Christopher-Hennings J, Gava D, Schaefer R, Caron L, Dee S, Diel DG. 2016. Detection of the emerging picornavirus Senecavirus A in Pigs, mice, and houseflies. J Clin Microbiol 54:1536–1545. doi:10.1128/JCM.03390-15 27030489 PMC4879313

[11] Li N, Qiao QL, Guo HF, Wang BY, Huang Q, Wang Z, Li YT, Zhao J. 2021. Evaluation of immunogenicity and protective efficacy of a novel Senecavirus A strain-based inactivated vaccine in mice. Res Vet Sci 142:133–140. doi:10.1016/j.rvsc.2021.12.010 34952258

[12] Segalés J, Barcellos D, Alfieri A, Burrough E, Marthaler D. 2017. Senecavirus A. Vet Pathol 54:11–21. doi:10.1177/0300985816653990 27371541

[13] Song J, Hou L, Quan R, Wang D, Jiang H, Liu J. 2022. Synergetic contributions of viral VP1, VP3, and 3C to activation of the AKT-AMPK-MAPK-MTOR signaling pathway for seneca valley virus-induced autophagy. J Virol 96:e0155021. doi:10.1128/JVI.01550-21 34757844 PMC8791279

[14] Cao L, Zhang R, Liu T, Sun Z, Hu M, Sun Y, Cheng L, Guo Y, Fu S, Hu J, Li X, Yu C, Wang H, Chen H, Li X, Fry EE, Stuart DI, Qian P, Lou Z, Rao Z. 2018. Seneca Valley virus attachment and uncoating mediated by its receptor anthrax toxin receptor 1. Proc Natl Acad Sci USA 115:13087–13092. doi:10.1073/pnas.1814309115 30514821 PMC6304951

[15] Li Z, Yang J, Ma R, Xie S, Wang D, Quan R, Wen X, Liu J, Song J. 2025. Seneca Valley virus 3C protease cleaves HDAC4 to antagonize type I interferon signaling. J Virol 99:e0217624. doi:10.1128/jvi.02176-24 39927774 PMC11915795

[16] Xue Q, Liu H, Zhu Z, Yang F, Ma L, Cai X, Xue Q, Zheng H. 2018. Seneca Valley Virus 3C^pro^ abrogates the IRF3- and IRF7-mediated innate immune response by degrading IRF3 and IRF7. Virology (Auckl) 518:1–7. doi:10.1016/j.virol.2018.01.028 29427864

[17] Nakamura N. 2018. Ubiquitin system. Int J Mol Sci 19:1080. doi:10.3390/ijms19041080 29617326 PMC5979459

[18] Wang W, Bi Z, Song S. 2023. Host E3 ligase Hrd1 ubiquitinates and degrades H protein of canine distemper virus to inhibit viral replication. Vet Res 54:30. doi:10.1186/s13567-023-01163-z 37009870 PMC10069049

[19] Li Z, Hao P, Zhao Z, Gao W, Huan C, Li L, Chen X, Wang H, Jin N, Luo ZQ, Li C, Zhang W. 2023. The E3 ligase RNF5 restricts SARS-CoV-2 replication by targeting its envelope protein for degradation. Sig Transduct Target Ther 8:53. doi:10.1038/s41392-023-01335-5 PMC9897159 36737599

[20] Fan W, Mar KB, Sari L, Gaszek IK, Cheng Q, Evers BM, Shelton JM, Wight-Carter M, Siegwart DJ, Lin MM, Schoggins JW. 2021. TRIM7 inhibits enterovirus replication and promotes emergence of a viral variant with increased pathogenicity. Cell 184:3410–3425. doi:10.1016/j.cell.2021.04.047 34062120 PMC8276836

[21] Meacham GC, Patterson C, Zhang W, Younger JM, Cyr DM. 2001. The Hsc70 co-chaperone CHIP targets immature CFTR for proteasomal degradation. Nat Cell Biol 3:100–105. doi:10.1038/35050509 11146634

[22] Narayan V, Landré V, Ning J, Hernychova L, Muller P, Verma C, Walkinshaw MD, Blackburn EA, Ball KL. 2015. Protein-protein interactions modulate the docking-dependent E3-ubiquitin ligase activity of carboxy-terminus of Hsc70-interacting protein (CHIP). Mol Cell Proteomics 14:2973–2987. doi:10.1074/mcp.M115.051169 26330542 PMC4638040

[23] Fan M, Park A, Nephew KP. 2005. CHIP (carboxyl terminus of Hsc70-interacting protein) promotes basal and geldanamycin-induced degradation of estrogen receptor-alpha. Mol Endocrinol 19:2901–2914. doi:10.1210/me.2005-0111 16037132

[24] Xiong W, Liu S, Cai W, Wen J, Fu Y, Peng J, Zheng Z. 2017. The carboxyl terminus of heat shock protein 70-interacting protein (CHIP) participates in high glucose-induced cardiac injury. Free Radic Biol Med 106:339–344. doi:10.1016/j.freeradbiomed.2017.02.047 28257878

[25] Chen Z, Barbi J, Bu S, Yang HY, Li Z, Gao Y, Jinasena D, Fu J, Lin F, Chen C, et al.. 2013. The ubiquitin ligase Stub1 negatively modulates regulatory T cell suppressive activity by promoting degradation of the transcription factor Foxp3. Immunity 39:272–285. doi:10.1016/j.immuni.2013.08.006 23973223 PMC3817295

[26] Yang M, Wang C, Zhu X, Tang S, Shi L, Cao X, Chen T. 2011. E3 ubiquitin ligase CHIP facilitates Toll-like receptor signaling by recruiting and polyubiquitinating Src and atypical PKC{zeta}. J Exp Med 208:2099–2112. doi:10.1084/jem.20102667 21911421 PMC3182058

[27] Wang S, Li Y, Hu YH, Song R, Gao Y, Liu HY, Shu HB, Liu Y. 2013. STUB1 is essential for T-cell activation by ubiquitinating CARMA1. Eur J Immunol 43:1034–1041. doi:10.1002/eji.201242554 23322406

[28] Shao M, Li L, Song S, Wu W, Peng P, Yang C, Zhang M, Duan F, Jia D, Zhang J, Wu H, Zhao R, Wang L, Ruan Y, Gu J. 2016. E3 ubiquitin ligase CHIP interacts with C-type lectin-like receptor CLEC-2 and promotes its ubiquitin-proteasome degradation. Cell Signal 28:1530–1536. doi:10.1016/j.cellsig.2016.07.007 27443248

[29] Zhang S, Zhang X, Bie Y, Kong J, Wang A, Qiu Y, Zhou X. 2022. STUB1 regulates antiviral RNAi through inducing ubiquitination and degradation of Dicer and AGO2 in mammals. Virol Sin 37:569–580. doi:10.1016/j.virs.2022.05.001 35533808 PMC9437610

[30] Yang X, Kong N, Qin W, Zhai X, Song Y, Tong W, Li L, Liu C, Zheng H, Yu H, Zhang W, Tong G, Shan T. 2023. PGAM5 degrades PDCoV N protein and activates type I interferon to antagonize viral replication. J Virol 97:e0147023. doi:10.1128/jvi.01470-23 37882521 PMC10688367

[31] Wang Y, Du S, Zhu C, Wang C, Yu N, Lin Z, Gan J, Guo Y, Huang X, He Y, Robertson E, Qu D, Wei F, Cai Q. 2020. STUB1 is targeted by the SUMO-interacting motif of EBNA1 to maintain Epstein-Barr Virus latency. PLoS Pathog 16:e1008447. doi:10.1371/journal.ppat.1008447 32176739 PMC7105294

[32] Wen W, Li X, Yin M, Wang H, Qin L, Li H, Liu W, Zhao Z, Zhao Q, Chen H, Hu J, Qian P. 2021. Selective autophagy receptor SQSTM1/ p62 inhibits Seneca Valley virus replication by targeting viral VP1 and VP3. Autophagy 17:3763–3775. doi:10.1080/15548627.2021.1897223 33719859 PMC8632295

[33] Kwon YT, Ciechanover A. 2017. The ubiquitin code in the ubiquitin-proteasome system and autophagy. Trends Biochem Sci 42:873–886. doi:10.1016/j.tibs.2017.09.002 28947091

[34] Liu Y, Zhou H, Tang X. 2023. STUB1/CHIP: new insights in cancer and immunity. Biomedicine & Pharmacotherapy 165:115190. doi:10.1016/j.biopha.2023.115190 37506582

[35] Soss SE, Rose KL, Hill S, Jouan S, Chazin WJ. 2015. Biochemical and proteomic analysis of ubiquitination of Hsc70 and Hsp70 by the E3 ligase CHIP. PLoS One 10:e0128240. doi:10.1371/journal.pone.0128240 26010904 PMC4444009

[36] Xu W, Marcu M, Yuan X, Mimnaugh E, Patterson C, Neckers L. 2002. Chaperone-dependent E3 ubiquitin ligase CHIP mediates a degradative pathway for c-ErbB2/Neu. Proc Natl Acad Sci USA 99:12847–12852. doi:10.1073/pnas.202365899 12239347 PMC130548

[37] Hou L, Zeng P, Wu Z, Yang X, Guo J, Shi Y, Song J, Zhou J, Liu J. 2024. Heat shock protein 70 enhances viral replication by stabilizing Senecavirus A nonstructural proteins L and 3D. Vet Res 55:158. doi:10.1186/s13567-024-01414-7 39695881 PMC11654094

[38] Song J, Wang D, Quan R, Liu J. 2021. Seneca Valley virus 3C^pro^ degrades heterogeneous nuclear ribonucleoprotein A1 to facilitate viral replication. Virulence 12:3125–3136. doi:10.1080/21505594.2021.2014681 34923914 PMC8923066

[39] Song J, Quan R, Wang D, Liu J. 2022. Seneca Valley Virus 3C^pro^ mediates cleavage and redistribution of nucleolin to facilitate viral replication. Microbiol Spectr 10:e0030422. doi:10.1128/spectrum.00304-22 35357201 PMC9045095

[40] Wang L, Prarat M, Hayes J, Zhang Y. 2016. Detection and genomic characterization of Senecavirus A. Emerg Infect Dis 22:1321–1323. doi:10.3201/eid2207.151897 27314491 PMC4918170

[41] Song J, Guo Y, Wang D, Quan R, Wang J, Liu J. 2024. Seneca Valley virus 3C protease cleaves OPTN (optineurin) to Impair selective autophagy and type I interferon signaling. Autophagy 20:614–628. doi:10.1080/15548627.2023.2277108 37930946 PMC10936645

[42] Patil G, Li S. 2019. Tripartite motif proteins: an emerging antiviral protein family. Future Virol 14:107–122. doi:10.2217/fvl-2018-0161 31406497 PMC6690340

[43] Zhou P, Ding X, Wan X, Liu L, Yuan X, Zhang W, Hui X, Meng G, Xiao H, Li B, Zhong J, Hou F, Deng L, Zhang Y. 2018. MLL5 suppresses antiviral innate immune response by facilitating STUB1-mediated RIG-I degradation. Nat Commun 9:1243. doi:10.1038/s41467-018-03563-8 29593341 PMC5871759

[44] Zhang Y, Hou P, He DC, Wang H, He H. 2021. RACK1 degrades MAVS to promote bovine ephemeral fever virus replication via upregulating E3 ubiquitin ligase STUB1. Vet Microbiol 257:109096. doi:10.1016/j.vetmic.2021.109096 33940459

[45] Zhao K, Zhang Q, Li X, Zhao D, Liu Y, Shen Q, Yang M, Wang C, Li N, Cao X. 2016. Cytoplasmic STAT4 promotes antiviral type I IFN production by blocking CHIP-mediated degradation of RIG-I. J Immunol 196:1209–1217. doi:10.4049/jimmunol.1501224 26695369

[46] Ali A, Farooqui SR, Banerjea AC. 2019. The host cell ubiquitin ligase protein CHIP is a potent suppressor of HIV-1 replication. Journal of Biological Chemistry 294:7283–7295. doi:10.1074/jbc.RA118.007257 30885946 PMC6509501

[47] Joazeiro CA, Weissman AM. 2000. RING finger proteins: mediators of ubiquitin ligase activity. Cell 102:549–552. doi:10.1016/s0092-8674(00)00077-5 11007473

[48] Clague MJ, Urbé S. 2010. Ubiquitin: same molecule, different degradation pathways. Cell 143:682–685. doi:10.1016/j.cell.2010.11.012 21111229

[49] Chen ZJ, Sun LJ. 2009. Nonproteolytic functions of ubiquitin in cell signaling. Mol Cell 33:275–286. doi:10.1016/j.molcel.2009.01.014 19217402

[50] Raiborg C, Stenmark H. 2009. The ESCRT machinery in endosomal sorting of ubiquitylated membrane proteins. Nature 458:445–452. doi:10.1038/nature07961 19325624

[51] Wang L, Zhang R. 2019. Towards computational models of identifying protein ubiquitination sites. Curr Drug Targets 20:565–578. doi:10.2174/1389450119666180924150202 30246637

[52] Wang K, Zou C, Wang X, Huang C, Feng T, Pan W, Wu Q, Wang P, Dai J. 2018. Interferon-stimulated TRIM69 interrupts dengue virus replication by ubiquitinating viral nonstructural protein 3. PLoS Pathog 14:e1007287. doi:10.1371/journal.ppat.1007287 30142214 PMC6126873

[53] Tsuchida T, Zou J, Saitoh T, Kumar H, Abe T, Matsuura Y, Kawai T, Akira S. 2010. The ubiquitin ligase TRIM56 regulates innate immune responses to intracellular double-stranded DNA. Immunity 33:765–776. doi:10.1016/j.immuni.2010.10.013 21074459

[54] Li P, Kurata Y, Maharani N, Mahati E, Higaki K, Hasegawa A, Shirayoshi Y, Yoshida A, Kondo T, Kurozawa Y, Yamamoto K, Ninomiya H, Hisatome I. 2015. E3 ligase CHIP and Hsc70 regulate Kv1.5 protein expression and function in mammalian cells. J Mol Cell Cardiol 86:138–146. doi:10.1016/j.yjmcc.2015.07.018 26232501

[55] Luo W, Zhong J, Chang R, Hu H, Pandey A, Semenza GL. 2010. Hsp70 and CHIP selectively mediate ubiquitination and degradation of hypoxia-inducible factor (HIF)-1alpha but Not HIF-2alpha. J Biol Chem 285:3651–3663. doi:10.1074/jbc.M109.068577 19940151 PMC2823506

[56] Wei Q, Sha Y, Bhattacharya A, Abdel Fattah E, Bonilla D, Jyothula SSSK, Pandit L, Khurana Hershey GK, Eissa NT. 2014. Regulation of IL-4 receptor signaling by STUB1 in lung inflammation. Am J Respir Crit Care Med 189:16–29. doi:10.1164/rccm.201305-0874OC 24251647 PMC3919125

[57] Sha Y, Rao L, Settembre C, Ballabio A, Eissa NT. 2017. STUB1 regulates TFEB-induced autophagy-lysosome pathway. EMBO J 36:2544–2552. doi:10.15252/embj.201796699 28754656 PMC5579343

[58] Yan Y, Wu L, Yuan Y, Wang H, Yin H, Li M, Chai L, Liang R, Liu Y, Zhao D, Xing J, Li P, Li X. 2023. Species-specific cleavage of cGAS by picornavirus protease 3C disrupts mitochondria DNA-mediated immune sensing. PLoS Pathog 19:e1011641. doi:10.1371/journal.ppat.1011641 37708231 PMC10521975

[59] Song J, Quan R, Wang D, Liu J. 2022. Seneca Valley virus 3C (pro) cleaves heterogeneous nuclear ribonucleoprotein K to facilitate viral replication. Front Microbiol 13:945443. doi:10.3389/fmicb.2022.945443 35875542 PMC9298500

[60] Zhang W, Li W, Yang Y, Cao W, Shao W, Huang M, Wang J, Chen Z, Cai J, Liu H, Zhao X, Dong X, Zhou T, Tian H, Zhu Z, Yang F, Zheng H. 2025. RING finger protein 5 is a key anti-FMDV host factor through inhibition of virion assembly. PLoS Pathog 21:e1012848. doi:10.1371/journal.ppat.1012848 39823440 PMC11741381

[61] Zhou P, Zhang Q, Yang Y, Liu D, Wu W, Jongkaewwattana A, Jin H, Zhou H, Luo R. 2025. TRIM14 restricts tembusu virus infection through degrading viral NS1 protein and activating type I interferon signaling. PLoS Pathog 21:e1013200. doi:10.1371/journal.ppat.1013200 40435148 PMC12118852

[62] Li H, Lin C, Qi W, Sun Z, Xie Z, Jia W, Ning Z. 2023. Senecavirus A-induced glycolysis facilitates virus replication by promoting lactate production that attenuates the interaction between MAVS and RIG-I. PLoS Pathog 19:e1011371. doi:10.1371/journal.ppat.1011371 37126525 PMC10174517

